# Proximal Remote Sensing to Non-destructively Detect and Diagnose Physiological Responses by Host Insect Larvae to Parasitism

**DOI:** 10.3389/fphys.2018.01716

**Published:** 2018-12-04

**Authors:** Christian Nansen, Michael R. Strand

**Affiliations:** ^1^Department of Entomology and Nematology, University of California, Davis, Davis, CA, United States; ^2^Department of Entomology, University of Georgia, Athens, GA, United States

**Keywords:** hyperspectral image classification, parasitization, reflectance, remote sensing, stress detecting, noninvasive method

## Abstract

As part of identifying and characterizing physiological responses and adaptations by insects, it is paramount to develop non-destructive techniques to monitor individual insects over time. Such techniques can be used to optimize the timing of when in-depth (i.e., destructive sampling of insect tissue) physiological or molecular analyses should be deployed. In this article, we present evidence that hyperspectral proximal remote sensing can be used effectively in studies of host responses to parasitism. We present time series body reflectance data acquired from individual soybean loopers (*Chrysodeixis includens*) without parasitism (control) or parasitized by one of two species of parasitic wasps with markedly different life histories: *Microplitis demolitor*, a solitary larval koinobiont endoparasitoid and *Copidosoma floridanum*, a polyembryonic (gregarious) egg-larval koinobiont endoparasitoid. Despite considerable temporal variation in reflectance data 1–9 days post-parasitism, the two parasitoids caused uniquely different host body reflectance responses. Based on reflectance data acquired 3–5 days post-parasitism, all three treatments (control larvae, and those parasitized by either *M. demolitor* or *C. floridanum*) could be classified with >85 accuracy. We suggest that hyperspectral proximal imaging technologies represent an important frontier in insect physiology, as they are non-invasive and can be used to account for important time scale factors, such as: minutes of exposure or acclimation to abiotic factors, circadian rhythms, and seasonal effects. Although this study is based on data from a host-parasitoid system, results may be of broad relevance to insect physiologists. Described approaches provide a non-invasive and rapid method that can provide insights into when to destructively sample tissue for more detailed mechanistic studies of physiological responses to stressors and environmental conditions.

## Introduction

In studies of basic insect physiology and evolutionary adaptations to environmental conditions and stressors, there is growing appreciation for the complex dynamics of physiological responses across different time scales. That is, a range of time scale factors can dramatically affect the magnitude and type of physiological responses by individual insects. Examples include: (1) minutes of exposureor acclimation to abiotic factors like temperature (Macdonald et al., 2004; Weidlich et al., 2012), UV irradiation (Meng et al., 2009), low oxygen levels (Deng et al., 2018); (2) when during circadian rhythms physiological processes are investigated (Danks, 2003, 2005; Weidlich et al., 2012; Bloch et al., 2013; Suszczynska et al., 2017; Nose et al., 2018); and 3) sampling time during field seasons (Danks, 2003, 2005; Matsuda et al., 2017). Time scale factors further vary among life stages and in response to biological stress factors such as infection or parasitism (Weidlich et al., 2012; Nose et al., 2018).

Parasitoids are free-living insects as adults whose offspring develop by feeding in or on the body of another arthropod. Most parasitoids complete their immature development by feeding on a single host and most hosts ultimately die as a consequence of being parasitized. For this reason, a number of parasitoids are used as biological control agents for management of important insect pest species. Parasitoids occur in several orders of insects but are most prevalent in the order Hymenoptera where estimates suggest more than 200,000 species exist (Godfray, 1994; Quicke, 1997). Most parasitoids are specialists that parasitize a restricted number of host species in a particular host life stage (eggs, larvae/nymphs, pupae, or adults) (Pennacchio and Strand, 2006). Parasitoids are often divided into idiobionts, whose hosts cease development after parasitism, and koinobionts, whose hosts continue to develop as the parasitoid’s offspring grow (Haeselbarth, 1979; Askew and Shaw, 1986). Parasitoids also are commonly divided into ectoparasitic species whose offspring grow by feeding externally on hosts or endoparasitoids whose offspring grow by feeding internally. Most known idiobionts are either ectoparasitoids that paralyze and lay eggs on the surface of larval stage hosts or are endoparasitoids that lay their eggs inside sessile host stages like eggs or pupae. *Microplitis demolitor* Wilkinson (Hymenoptera: Braconidae) is a solitary larval koinobiont endoparasitoid that dramatically reduces host growth due to factors including venom, a polydnavirus and teratocytes this species introduces into its host (Strand, 2014; Strand and Burke, 2014). Due to parasitism-induced inhibition of growth, it is easy to differentiate soybean loopers parasitized by *M. demolitor* from non-parasitized larvae as long as the developmental stage of the host larva is known. In addition, a single *M. demolitor* offspring emerges from the host larva 7–9 days post-parasitism to pupate, while non-parasitized larvae continue to increase in size to the final instar, which is followed by spinning of a silken cocoon and pupation (Strand, 1990; Strand and Noda, 1991). *Copidosoma floridanum* (Ashmead) (Hymenoptera: Encyrtidae) is a polyembryonic (gregarious) egg-larval koinobiont endoparasitoid that minimally alters host growth until late in the final instar, when thousands of wasp progeny complete their development (Ode and Strand, 1995; Giron et al., 2007). Distinguishing soybean loopers parasitized by *C. floridanum* from non-parasitized larvae based on morphology is very difficult until the ultimate (final) instar due to parasitized hosts exhibiting few differences in size or growth rates. However, *C. floridanum* parasitized larvae grow to a larger than non-parasitized larvae by the final instar (Ode and Strand, 1995). After the host spins a silken cocoon, *C. floridanum* larvae consume all internal organs and pupate inside the host’s exoskeleton to form a “mummy,” whereas non-parasitized larvae pupate inside of their silken cocoon to later emerge as an adult moth (Ode and Strand, 1995). Development of non-destructive methods for distinguishing parasitized from non-parasitized larval hosts, and also diagnosis of the parasitoid species, could be highly useful in both basic studies of parasitoid biology and pest management where estimates of parasitism rates can affect control decisions.

Proximal remote sensing is a fast and non-invasive imaging technique, in which target objects, such as insects, are placed within short distance under a camera lens for only a few seconds and reflectance or transmittance data are acquired with an imaging sensor. The imaging sensor can have three spectral bands (normally with the visible light divided into three regions: red, green, and blue), multi-spectral (typically with 5–12 spectral bands), or hyperspectral (with >50 spectral bands. Recent reviews describe the applications of proximal remote sensing in studies of insects (Nansen, 2016; Nansen and Elliott, 2016). Although most entomological applications of proximal remote sensing focus on identification of insects or plant responses to insect herbivory, there are several physiological studies, in which proximal remote sensing of insect body reflectance was used. These include: (1) age-grading of mosquitoes (*Anopheles* spp.) (Sikulu et al., 2010; Sikulu et al., 2014), biting midges (*Culicoides sonorensis*) (Reeves et al., 2010), and two species of fruit flies (*Drosophila melanogaster* and *D. simulans*) (Aw et al., 2012), (2) infection status of insects by intracellular bacteria in the genus *Wolbachia* (Aw et al., 2012), (3) mating status of honeybee queens (Webster et al., 2009); (4) ontogeny of puparia of two blowfly species (Voss et al., 2016), and (5) “terminal stress” responses caused exposure of maize weevils (*Sitophilus zeamais*) to insecticidal plant extracts and infection of darkling beetles (*Cynaus angustus*) by entomopathogenic nematodes (Nansen et al., 2015). Only two studies have previously used hyperspectral proximal remote sensing in parasitism of insects: (1) distinction of species of *Trichogramma* immatures developing inside parasitized host eggs (Nansen et al., 2014a) and (2) distinguishing non-parasitized fruit fly pupae (nine different species) from pupae parasitized by the wasp *Pachycrepoideus vindemiae* (Rondani) (Pteromalidae) (Nansen, 2016). Despite the steadily growing number of studies, in which proximal remote sensing technologies have been used in studies of insects, associations between body reflectance signals and underlying physiological processes are largely unknown. Although based on a study of plants, a recently published study characterized associations between leaf reflectance and phytocompounds (Ribeiro et al., 2018).

In this study, we used proximal remote sensing to acquire time series reflectance data from individual soybean looper [*Chrysodeixis includens* Walker (Lepidoptera: Noctuidae)] larvae, which were either non-parasitized (control) or parasitized by *M. demolitor* or *C. floridanum*. Due to the distinct differences in life histories of *M. demolitor* and *C. floridanum*, the key questions addressed in this study were: (1) when is the earliest time point post-parasitism that parasitized larvae can be accurately distinguished from non-parasitized larvae, and (2) do reflectance data acquired from the body of host larvae enable accurate differentiation of parasitism by a solitary versus gregarious koinobiont endoparasitoid? We discuss the result-derived answers to these questions in the context of existing molecular and physiological knowledge about the host’s immune responses.

## Materials and Methods

### Insects

Soybean loopers were individually reared on an artificial diet at 27°C as previously described (Strand, 1990) in plastic cups containing enough food for non-parasitized hosts to pupate or parasitized hosts to produce wasp offspring. Soybean loopers were parasitized by *M. demolitor* as day 1 s instars and as day 1 eggs by *C. floridanum* (Strand and Noda, 1991; Ode and Strand, 1995). Hosts were individually parasitized to assure that each host was only parasitized once by a single wasp female. Proximal remote sensing data were acquired from individual larvae in the following three treatments: (1) non-parasitized (referred to as “control”) soybean loopers, (2) soybean loopers parasitized by a solitary koinobiont endoparasitoid, *M. demolitor*, and (3) soybean loopers parasitized by a gregarious koinobiont endoparasitoid, *C. floridanum*.

Initially, 28–30 replicated soybean loopers were included for each treatment, and proximal remote sensing time series data were acquired daily for 9 consecutive days post-parasitism. Some of the soybean loopers, especially those assigned to parasitism by *C. floridanum*, died during the 9 days of data acquisition. In addition, not all host larvae exposed to parasitism were actually parasitized. The latter was confirmed by rearing individual larvae until they pupated (control) or produced parasites (a single *M. demolitor* pupa or larvae forming a mummy containing thousands of *C. floridanum*). Consequently, the total number of observations was 605 (control = 376, *M. demolitor* = 123, and *C. floridanum* = 107). During the 9 days of proximal remote sensing data acquisition, host larvae were maintained under standard laboratory conditions (20–22^o^C and 30–40% RH).

### Hyperspectral Proximal Remote Sensing

**FIGURE 1:**
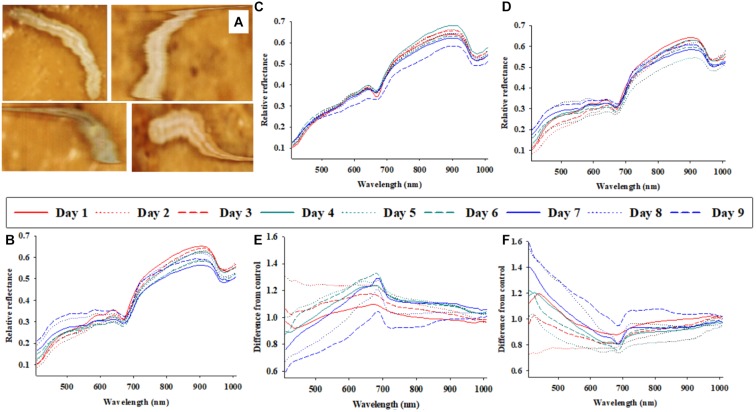
Representative photos of soybean loopers (*Chrysodeixis includens*) during acquisition of proximal remote sensing data **(A)**. Daily (1–9 days of parasitism) average soybean looper body reflectance profiles from three treatments: control (non-parasitized) **(B)**, parasitized by *Microplitis demolitor* **(C)**, parasitized by *Copidosoma floridanum* **(D)**. The effects of parasitism by *M. demolitor* **(E),** or *C. floridanum* **(F)** are also presented as differences from control larvae.

The acquisition of proximal remote sensing data was truly non-invasive as soybean loopers where not removed from their rearing cups (Figure 1A). The time to acquire proximal remote sensing data varied somewhat among soybean loopers (occasionally, the acquisition had to be repeated because of extensive movement by the larvae), but the vast majority of hyperspectral images from each larva was acquired within 30 s, which minimized exposure of test animals to stress from the lamp required for image acquisition.

Similar to previously published studies (Nansen, 2011, 2012; Nansen et al., 2013a, 2014b; Zhao et al., 2014; Zhang et al., 2015), we used a hyperspectral push broom spectral camera (PIKA II, Resonon Inc., Bozeman, MT, United States). The objective lens had a 35 mm focal length (maximum aperture of F1.4) and was optimized for the visible and NIR spectra. The main specifications of the spectral camera are as follows: interface, Firewire (IEEE 1394b); output, digital (14 bit); 240 spectral bands (from 383 to 1036 nm) by 1600 pixels (spatial); angular field of view of 7^0^; and spectral resolution of approximately 2.1 nm. All hyperspectral images were collected with artificial lighting from 15W, 12V LED light bulbs mounted in two angled rows, one on either side of the lens, with three bulbs in each row. A voltage stabilizer (Tripp-Lite, PR-7b^1^) powered the lighting. Ambient climate conditions for data acquisition were between 19 and 22°C and between 30 and 40% relative humidity. A piece of white Teflon (K-Mac Plastics, MI, United States) was used for white calibration, and “relative reflectance” refers to proportional reflectance compared with that obtained from Teflon. Consequently, relative reflectance values ranged from 0 to 1. Hyperspectral images were acquired at a spatial resolution of about 45 pixels mm^2^.

Prior to analysis, we excluded the first and last nine of the original 240 spectral bands, and the remaining 222 spectral bands (from 408 to 1011 nm) were spectrally 3X-binned (averaged) into 74 spectral bands. The spectral binning represents a loss of spectral resolution, but it was performed to ensure that the number of observations (average reflectance profiles from larvae) far exceeded the number of spectral bands. Moreover, model over-fitting due to the Hughes phenomenon or violation of the principle of parsimony (Hawkins, 2004) is a major concern when the number of explanatory variables is similar or exceeds the number of observations (Kemsley, 1996; Defernez and Kemsley, 1997; Nansen et al., 2013a; Nansen and Elliott, 2016).

### Experimental Design and Data Analysis

“Robustness” or repeatability of proximal remote sensing data is a major technical challenge (Peleg et al., 2005; Nansen, 2011; Nansen and Elliott, 2016). Low robustness implies a high level of variability of proximal remote sensing data acquired from: (1) the same object at multiple time points, (2) different portions of the same object, (3) several objects in the same category or class. For instance, in this study, we acquired time series data to detect and diagnose the stress imposed by parasitoids, when at the same time the host larvae are growing over time. In other words, the body reflectance profiles of control (non-parasitized) larvae are changing over time in response to ontogeny, and this ontogeny-driven change in host body reflectance has to be separated from unique reflectance responses to parasitism.

Data processing and analyses were conducted in PC-SAS 9.4 (SAS Institute, NC, United States), and all statistical analyses were based on average host body reflectance profiles from individual larvae. Two data analyses were performed with average host body reflectance profiles as explanatory variables: (1) For each of the three treatments (three separate analyses), days of parasitism was used as response variable, and (2) the data was grouped into seven 3 days intervals: 1–3, 2–4, 3–5, 4–6, 5–7, 6–8, and 7–9 days, and treatment [1) non-parasitized (control) soybean loopers, (2) soybean loopers parasitized by *M. demolitor*, and (3) soybean loopers parasitized by *C. floridanum* was used as response variable. For both analyses, we performing a linear discriminant classifications (proc discrim) (Fisher, 1936). Initially, stepwise linear discriminant analysis (proc stepwise) was used to only select the spectral bands (out of the total of 74 spectral bands) with significant contribution to each of the linear discriminant classification models. The selected subset of explanatory variables was used to generate the linear discriminant classification models for each of the seven time intervals of parasitism, and the classification accuracy of each linear discriminant model was quantified on the basis of 80% of the data being randomly selected as training data set and the remaining 20% of the data used for independent validation. This validation procedure was repeated 10 times to calculate average accuracies for each linear discriminant classification model.

## Results

Figure 1A shows four representative images (as acquired with the hyperspectral camera) of soybean loopers, when imaged non-invasively while moving around inside a plastic petri dish on top of food media. The hyperspectral camera used in this study is a line-scanning device, which means that each image is composed of a sequence of individual lines (frames) that are captured as the camera scans across the object (in this case a plastic petri dish). A hyperspectral image of a single soybean looper was acquired within about 10 s. As the larvae moved around, the hyperspectral images of individual larvae become slightly distorted, but it is still possible to identify pixels representing larval body and differentiate them from the background (food media).

### Temporal Trends in Average Body Reflectance Profiles

**FIGURE 2:**
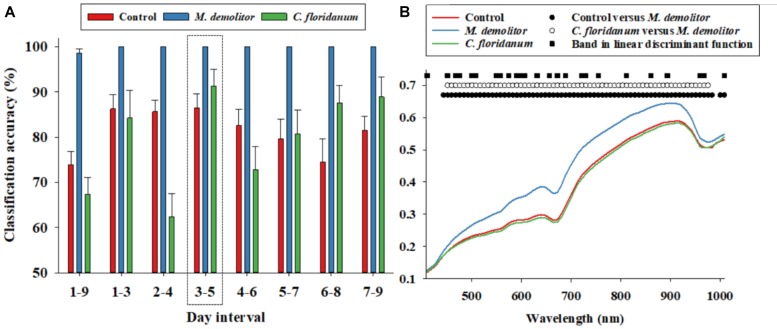
Daily (1–9 days of parasitism) proximal remote sensing data were divided into 3 days intervals, and a linear discriminant analysis was performed for each 3 days interval to classify soybean loopers from the three treatments (control, parasitized by *Microplitis demolitor* or parasitized by *Copidosoma floridanum*). Classification accuracies (based on independent validation) is presented **(A)**. Average soybean looper body reflectance profiles after 3–5 days post-parasitism are presented for the three treatments **(B)**. We performed an analysis of variance followed by a Tukey–Kramer *post hoc* test for all 74 spectral bands. Black circles indicate significant difference in average reflectance between control and *M. demolitor* parasitized larvae. White circles denote significant difference in average reflectance between control and *C. floridanum* parasitized larvae. Black squares denote spectral bands selected and included in the linear discriminant analysis of average reflectance profiles from the three treatments.

Figures 1B–D show the average host body reflectance profiles over 9 consecutive days for: (1) control soybean loopers (Figure 1B), (2) soybean loopers parasitized by *M. demolitor* (Figure 1C), and (3) soybean loopers parasitized by *C. floridanum* (Figure 1D). Each daily curve represents the average of 9–24 soybean loopers, and it is seen that, for all three treatments, there was considerable variation in body reflectance, especially in spectral bands within the range from 700 to 900 nm. In addition, in spectral bands from 408 to 600 nm, the average reflectance from non-parasitized soybean loopers remained fairly constant during the 9 days compared to average reflectance from parasitized soybean loopers.

In Figures 1E,F, the daily average host body reflectance profiles from larvae parasitized by *M. demolitor* or *C. floridanum* were divided with average host body reflectance profiles from control larvae on the same day. Consequently, these two figures illustrate the relative effect of parasitism for each of the 9 days, and: *y*-value < 1 implies that parasitism caused a decrease in larval body reflectance, *y*-value > 1 implies that parasitism caused an increase in larval body reflectance, and *y*-value close to 1 implies that parasitism had negligible effect on larval body reflectance. Parasitism by *M. demolitor* (Figure 1E) caused a decrease in reflectance in spectral bands from 410 to 450 nm 7–9 days post-parasitism, and it also caused an increase in reflectance in spectral bands near 680 nm 3–7 days post-parasitism. Parasitism by *C. floridanum* (Figure 1F) caused an increase in reflectance in spectral bands from 408 to 450 nm 7–9 days post-parasitism, and it also caused a decrease in reflectance in spectral bands near 680 nm 3–7 days post-parasitism.

Average reflectance profiles in Figure 1, presented either as actual reflectance profiles or as relative to those from control larvae, were included to underscore an important challenge associated with analyses of time series of reflectance data acquired from living organisms (seeds, leaves, insects, etc.): that the data acquired from control organisms are non-constant, so the “baseline” is constantly changing. This obvious, but very important phenomenon, introduces considerable stochastic noise and it makes the identification of treatment effects more challenging. Despite the variation imposed by larval ontogeny, it appeared that solitary and gregarious koinobiont parasitoids caused somewhat opposite host body reflectance responses, and that these responses could be detected 3–7 days post-parasitism.

### Linear Discriminant Analyses of Temporal Trends in Host Body Reflectance Data

To examine the temporal variability in daily proximal remote sensing data in detail, we conducted linear discriminant analysis of each of the three treatments separately, in which day post-parasitism was used as explanatory variable. Tables 1–3 show the classification results from validations of each classification model, and it was shown that: (1) the day of parasitism could be predicted with 39–100% accuracy, (2) early instars were only vary rarely misclassified as late instar larvae, (3) late instars were not misclassified as early instars, and (4) middle-aged early instars showed the widest spread of misclassification. All of these trends would be expected under the assumption of ontogeny markedly influencing the proximal remote sensing data. Based on the results presented in Tables 1–3, we grouped the data into 3 days time intervals (1–3, 2–4, 3–5, 4–6, 5–7, 6–8, and 7–9 days – as indicated by 3 by 3 cell rectangles). In Tables 1–3, it is seen that these day intervals capture most of the misclassification, so this was considered a reasonable grouping of the proximal remote sensing data.

**Table 1:** Classification accuracy (%) of control (non-parasitized) soybean loopers (*Chrysodeixis includens*).

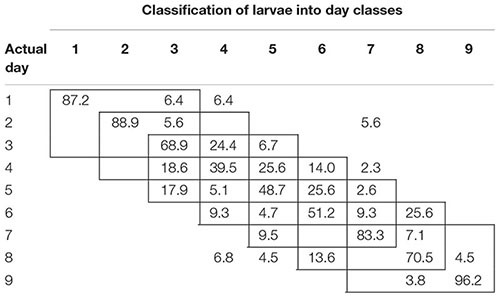

*Linear discriminant analyses were performed for individual data sets acquired 1–9 days after parasitism. Bold rectangles illustrate 3 days time intervals.*

**Table 2:** Classification accuracy (%) of soybean loopers (*Chrysodeixis includens*) parasitized by *Microplitis demolitor.*

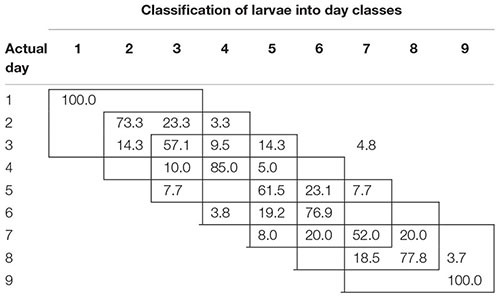

*Linear discriminant analyses were performed for individual data sets acquired 1–9 days after parasitism. Bold rectangles illustrate 3 days time intervals.*

**Table 3:** Classification accuracy (%) of soybean loopers (*Chrysodeixis includens*) parasitized by *Copidosoma floridanum.*

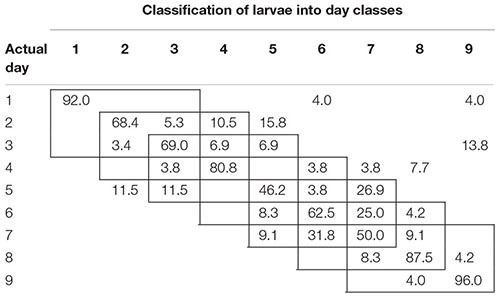

*Linear discriminant analyses were performed for individual data sets acquired 1–9 days after parasitism. Bold rectangles illustrate 3 days time intervals.*

### Linear Discriminant Analyses of Response to Parasitism

We used treatment (control, *M. demolitor*, or *C. floridanum*) as response variables, and replicated validations of the linear discriminant models showed that control larvae and those parasitized by *C. floridanum* could be classified with 65–75% accuracy, when all data from 1 to 9 days post-parasitism were included in the linear discriminant analysis (Figure 2A). This low level of classification accuracy was due to control larvae being misclassified as those parasitized by *C. floridanum* and vice versa. For comparison, when all data from 1 to 9 days post-parasitism were combined, host larvae parasitized by *M. demolitor* could be detected with >98% accuracy.

We suspected that the low classification accuracy of control larvae and those parasitized by *C. floridanum* was at least partially due to temporal variability in the body reflectance data, so the nine days post-parasitism were divided into seven 3 days intervals, and reflectance data from these were analyzed separately, and it was seen that the average classification accuracy of Figure 2A: (1) control larvae exceeded 80% when based on data from 1 to 6 days post-parasitism, (2) larvae parasitized by *M. demolitor* exceeded 99% for all seven 3 days intervals, and (3) larvae parasitized by *C. floridanum* varied considerably along the 9 days post-parasitism but exceeded 90% 3–5 days post-parasitism. As all three treatments were classified with >85% accuracy for reflectance data acquired 3–5 days post-parasitism, this was considered the most suitable time interval for detection and diagnosis of parasitism. Figure 2B shows the average reflectance profiles from the three treatments 3–5 days post-parasitism, and it also shows the spectral bands that were selected and used in the linear discriminant function to accurately separate the three treatments. It is seen that the spectral bands included in the linear discriminant function were fairly evenly distributed within the examined spectrum.

## Discussion

Reflectance data acquired from the body of individual soybean loopers were used to address the following two questions: (1) when is the earliest time point post-parasitism that parasitized larvae can be accurately distinguished from non-parasitized larvae, and (2) do reflectance data acquired from the body of host larvae enable accurate differentiation of parasitism by a solitary or a gregarious koinobiont endoparasitoid? The results from this study demonstrated that a solitary (*M. demolitor*) and a gregarious koinobiont (*C. floridanum*) parasitoid have opposite effects on host body reflectance in spectral bands from 408 to 450 nm and in spectral bands near 680 nm. This result is both surprising and important, because it implies that “parasitism” *per se* may not be detectable, if a group of host larvae are parasitized by a complex of parasitoid species and afterward grouped as parasitized and non-parasitized. The reason being that unique reflectance responses to one parasitoid may be out-weighed by reflectance responses to another parasitoid species. This phenomenon was observed in this study, in which parasitism by a solitary and a gregarious koinobiont parasitoid caused somewhat opposite host body reflectance responses at 408–450 nm and near 680 nm 3–7 days post-parasitism. Consequently, our results suggest that hyperspectral imaging is potentially sensitive and reliable enough to differentiate physiological responses by host larvae to different parasitoid species and/or to parasitoids with different life histories. As noted in the Introduction, prior studies established that *M. demolitor* causes almost immediate physiological responses in host larvae. Consistent with these alterations, host body reflectance data distinguished hosts parasitized by *M. demolitor* with >98% accuracy when data from 1 to 9 days post-parasitism were combined. So regarding *M. demolitor*, reflectance-based detection of parasitism was possible within a few days of parasitism and with very high degree of accuracy. The physiological responses could reflect that hosts parasitized by *M. demolitor* exhibit very rapid changes in feeding behavior which correlate with reduced weight gain due to alterations in nutrient availability and delays in molting (Pruijssers et al., 2009). It also is not surprising that reflectance-based detection of parasitism by *C. floridanum* was associated with lower classification accuracy. These findings underscore that parasitism by *C. floridanum* cause negligible effects on growth and molting in the early phases of parasitism relative to non-parasitized control larvae (Strand et al., 1992; Baehrecke et al., 1993; Ode and Strand, 1995). However, the results presented here do demonstrate that parasitism by *C. floridanum* could be detected with >85% accuracy if based on reflectance data acquired 3–5 days post-parasitism.

Although reflectance data were acquired from the external surface of insect larvae (or other objects), it is well-described in the medical literature that radiometric energy in the range from 600 to 1300 nm penetrates deeply into soft human tissues (including brain, liver, lung, and skin) (Wilson and Jacques, 1990). Consequently, the absorption and scattering properties as well as penetration depth of radiometric energy in the range from 600 to 1300 nm is used in the medical field as part of therapeutic dosimetry and diagnostic spectroscopy (Jacques, 1989; Wilson and Jacques, 1990; Stolik et al., 2000; Bargo, 2003). In addition, it has been shown that the penetration depth of radiometric energy into fruits and vegetables is a valuable indicator of their quality and therefore physiological state (Lammertyn et al., 2000; Nicolaï et al., 2007). Proximal remote sensing was also used to non-invasively determine and characterize the ontogeny of fly pupae (Voss et al., 2016). Finally, a recent experimental study based on proximal remote sensing of different objects [pieces of candy (Skittles), sheets of paper, magnolia leaves, and mosquito eggs] clearly showed how the acquired reflectance signal is highly influenced by the physical structure and biochemical composition of both the given object’s surface, the underlying tissues, and the thickness and size of objects (Nansen, 2018). The rather heterogeneous body of literature mentioned above is relevant to the current study, because of the important denominator that proximal remote sensing data acquired from the surface of objects provide partial insight into the composition and structure of below-surface tissues. Thus, it appears plausible to assume that internal physiological responses to parasitism can be detected and diagnosed on the basis of proximal remote sensing. In addition, it seems reasonable to speculate that general physiological variables, such as, larval content of water, carbohydrates, lips, protein, could partially explain why host body reflectance changes in response to parasitism. We are only aware of a few studies, in which reflectance data were directly associated with specific constituents of the imaged objects, and all of those studies were based on proximal remote sensing of plants (Nansen et al., 2013b; Lacoste et al., 2015; Ribeiro et al., 2018). Thus, future entomological research is needed, in which physiological, histological, and biochemical analyses are combined with acquisition of proximal remote sensing data from insects under different treatment regimes, including parasitism.

The growing appreciation for and adoption of proximal remote sensing is likely driven by the ability to, non-invasively and with only minor disturbance, acquire detailed spectral data over time from living animals assigned treatment groups. As an example with high relevance the to the current study, Nansen et al. (2015) acquired time series data to study insect responses to two terminal stressors: (1) adult weevils were maintained in petri dishes with maize kernels either untreated or treated with a plant-derived insecticide, and (2) adult darkling beetles were maintained in petri dishes with either control soil or soil inoculated with entomopathogenic nematodes. For both experiments, it was demonstrated that there was a significant change in adult insect body reflectance at time points, which coincided with published exposure times and known physiological responses by the insects to each of the two killing agents. An interesting result from this study was that the strongest insect body reflectance response was observed in spectral bands from 434 to 550 nm, and that: (1) exposure of adult weevils to a plant-derived insecticide caused a significant decrease in body reflectance, while (2) exposure of adult darkling beetles to a soil with entomopathogenic nematodes caused a significant increase in body reflectance. Moreover, the study highlighted a particular spectral region (434–550 nm) as a target for future and more in-depth studies of the relationship between host body reflectance and insect responses to terminal stressors. In addition, the study showed that two very different terminal stressors elicited either an increase or decrease in host body reflectance. The latter result suggests that proximal remote sensing data may be used to detect and diagnose insect responses to stressors. This particular study is highly relevant to the results presented in the current study, as we found that: (1) spectral bands in the same region (408–600 nm) responded strongly to a killing agent (in this case parasitism), and (2) the host larvae showed opposite reflectance responses to two parasitoids with markedly different life histories.

The main conclusions from the current study are: (1) larvae exposed to markedly different treatments (in this case, two parasitoids with different life histories) showed unique host body reflectance responses, and (2) despite considerable temporal variability, it was possible to detect clear trends and also identify a time interval (3–5 days of parasitism), in which the classification accuracy of proximal remote sensing data was higher than in other time intervals. In other words, time scale factors are of paramount importance, as the ability to classify objects (in this case parasitized larvae) vary over time. The latter conclusion implies that proximal remote sensing technologies can be used to monitor organismal responses over time and potentially identify time intervals with pronounced changes in host body reflectance. Such time intervals with pronounced changes in host body reflectance could be used to optimize the timing of when in-depth (i.e., destructive sampling of insect tissue) physiological or molecular analyses should be deployed. In other words, we argue that proximal remote sensing data analyses can be highly complementary to comprehensive physiological and molecular studies and be used to: (1) exclude outliers and reduce within-category variation among individuals, and (2) optimize when tissue should be sampled for costly and time-consuming physiological and molecular methods.

## Author Contributions

CN and MS contributed equally to the writing. CN collected and analyzed the proximal remote sensing data.

## Conflict of Interest Statement

The authors declare that the research was conducted in the absence of any commercial or financial relationships that could be construed as a potential conflict of interest.

## Footnotes

1 www.radioreference.com
